# Induction and suppression of gene silencing in plants by nonviral microbes

**DOI:** 10.1111/mpp.13362

**Published:** 2023-07-12

**Authors:** Eric Parperides, Kaoutar El Mounadi, Hernan Garcia‐Ruiz

**Affiliations:** ^1^ Department of Plant Pathology and Nebraska Center for Virology University of Nebraska‐Lincoln Lincoln Nebraska USA; ^2^ Department of Biology Kutztown University of Pennsylvania Kutztown Pennsylvania USA

**Keywords:** gene silencing, plant pathogens, plant symbionts, plant–microbe interactions, silencing suppressors, small interfering RNAs, trans‐kingdom movement of small RNAs

## Abstract

Gene silencing is a conserved mechanism in eukaryotes that dynamically regulates gene expression. In plants, gene silencing is critical for development and for maintenance of genome integrity. Additionally, it is a critical component of antiviral defence in plants, nematodes, insects, and fungi. To overcome gene silencing, viruses encode effectors that suppress gene silencing. A growing body of evidence shows that gene silencing and suppression of silencing are also used by plants during their interaction with nonviral pathogens such as fungi, oomycetes, and bacteria. Plant–pathogen interactions involve trans‐kingdom movement of small RNAs into the pathogens to alter the function of genes required for their development and virulence. In turn, plant‐associated pathogenic and nonpathogenic microbes also produce small RNAs that move trans‐kingdom into host plants to disrupt pathogen defence through silencing of plant genes. The mechanisms by which these small RNAs move from the microbe to the plant remain poorly understood. In this review, we examine the roles of trans‐kingdom small RNAs and silencing suppressors produced by nonviral microbes in inducing and suppressing gene silencing in plants. The emerging model is that gene silencing and suppression of silencing play critical roles in the interactions between plants and their associated nonviral microbes.

## Graphical abstract

We review the roles of trans‐kingdom small RNAs and silencing suppressors produced by nonviral microbes in inducing and suppressing gene silencing in plants as gene silencing/suppression plays a critical role in their interactions.

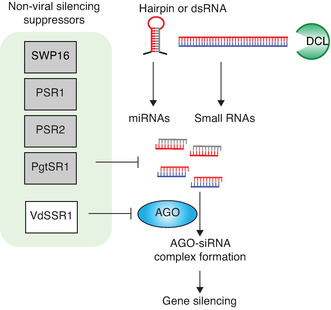

## 1 INTRODUCTION

As sessile organisms, plants encounter a wide variety of microbes over their lifespan. These microbes range from beneficial symbionts to dangerous pathogens such as viruses, viroids, bacteria, fungi, oomycetes, and nematodes. Modern large‐scale farming practices, such as monocropping of genetically identical plants, have led to the evolution of highly effective pathogens that cause $220 billion in losses each year (Chakraborty & Newton, 2011). In an effort to minimize loss of world food supply, a significant amount of research has focused on understanding the molecular interactions between plants and microbes. The relationship between plants and pathogens is based on a continuous evolutionary race between pathogenic effector proteins and plant resistance (R) genes (Kushalappa et al., 2016). In this relationship, pathogens mutate or rapidly evolve new sets of effector proteins capable of disrupting and exploiting the host immune response while plants evolve new R genes that detect and respond to those effectors (Michelmore et al., 2013).

**FIGURE 1:**
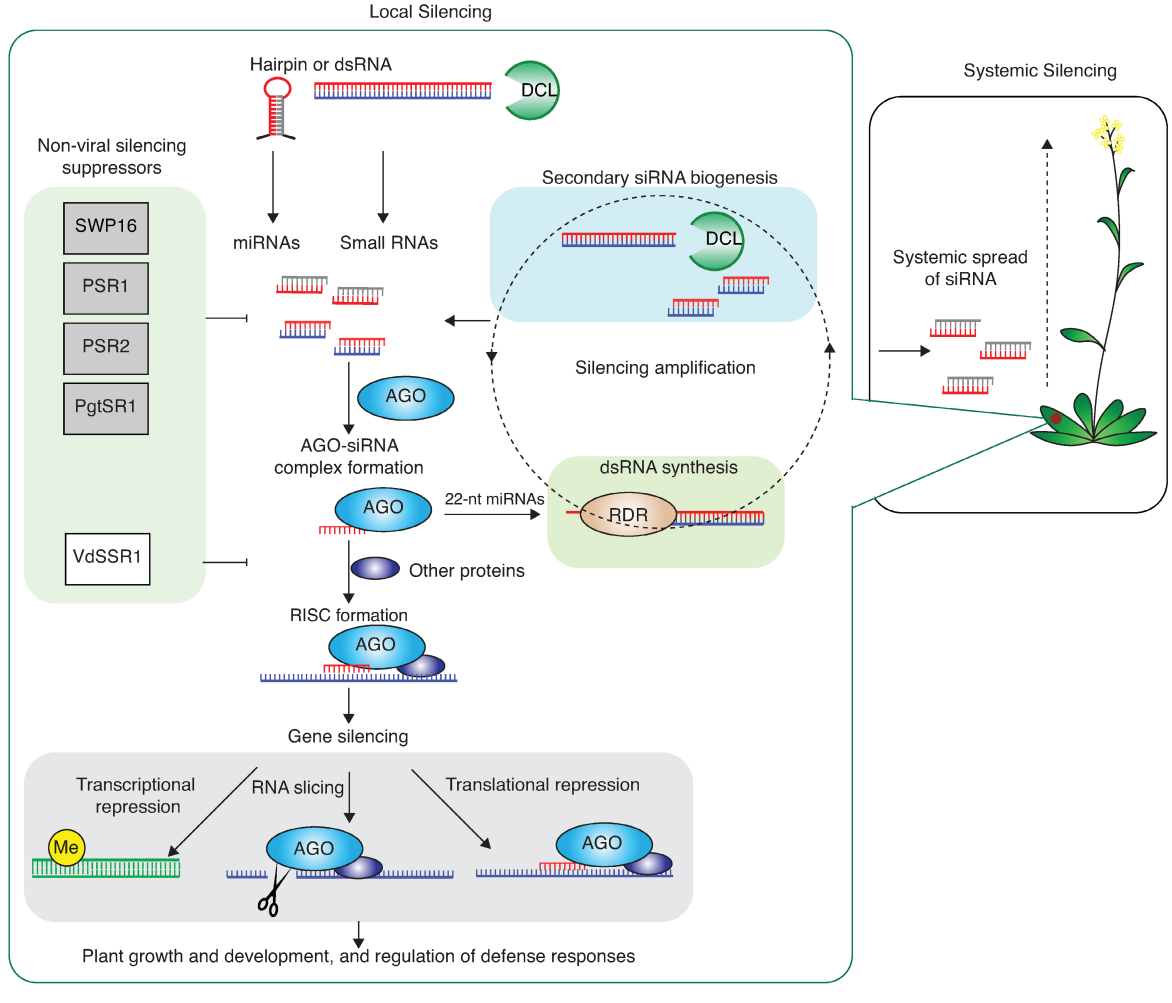
Silencing suppressors of nonviral plant pathogens. The diagram illustrates the general gene silencing pathway in plants initiated by double‐stranded (ds) RNA, the biogenesis of primary and secondary small interfering RNAs (siRNAs), RNA‐induced silencing complex (RISC) formation, and gene silencing by transcriptional repression, RNA slicing, or translational repression. Biogenesis of secondary siRNAs is triggered by some 22‐nucleotide (nt) micro‐RNAs (miRNAs) and includes the formation of dsRNA by cellular RNA‐dependent RNA polymerases. Systemic silencing involves the systemic spread of small interfering RNAs (siRNAs). Nonviral silencing suppressors described to date interfere with the biogenesis or activity of endogenous plant siRNAs. VdSSR1 inhibits the nuclear export of AGO1–miRNA complexes.

After pathogens are recognized by R genes through microbe‐associated molecular patterns (MAMPs), or effector proteins, downstream events such as gene silencing regulate gene expression and plant immunity. Gene silencing is conserved in eukaryotes. It is initiated by the presence of double‐stranded (ds) RNA in the plant cell. In the case of micro‐RNAs (miRNAs), single‐stranded RNA with a hairpin loop is encoded by host genes and is sufficient for initiation of the pathway. The ds pre‐miRNAs are diced into 21–22‐nucleotide (nt) fragments by Dicer‐like protein 1 (DCL1) (Kong et al., 2022). The resulting miRNAs are then loaded into argonaute (AGO) proteins and form part of the RNA‐induced silencing complex (RISC) that silences genes by slicing mRNA targets and inducing translational repression or DNA methylation. Interestingly, some miRNAs (22 nt long) trigger the biogenesis of secondary small interfering RNAs (siRNAs) through formation of dsRNAs by cellular RNA‐dependent RNA polymerases (Figure 1). In the process, miRNAs function as guide sequences by binding to complementary sequences on RNA targets (Zeng et al., 2019). Gene silencing is a critical component of antiviral immunity in plants (Yang & Li, 2018). Research has shown that gene silencing is important in plant–bacterial interactions, plant–fungal interactions, plant–oomycete interactions, and potentially plant–nematode interactions (Walsh et al., 2017).

RNA silencing plays a critical role in antiviral defence (Baulcombe, 2022). Biogenesis and function of virus‐derived siRNAs are similar to those of endogenous siRNAs in the host, and their genetic determinants partially overlap. During viral entry into the host cells, Dicer‐like endonucleases (DCLs) generate siRNA from viral genomes (Garcia‐Ruiz et al., 2010). These small RNAs (sRNAs) are used as guides by AGO proteins to specifically identify target viral RNAs for slicing or for biogenesis of dsRNAs by RNA‐dependent RNA polymerases (Wang et al., 2010). The resulting dsRNA is then diced into secondary siRNAs by DCL proteins, thus amplifying the strength of the silencing signal. siRNAs are not only localized to initially infected plant cells. Instead, they spread between cells via the plasmodesmata and systemically through the vascular system, triggering systemic silencing (Maizel et al., 2020) (Figure 1). This system of silencing and amplification provides a proactive layer of protection against further infection with the same virus.

To counter the potent defence by the plant's gene silencing system, plant viruses encode a unique class of proteins known as silencing suppressors that disrupt gene silencing at key steps of the pathway. Each viral genus carries at least one silencing suppressor protein required to establish viral infection, replication, and movement. One example is the helper component proteinase (HC‐Pro) from the genus *Potyvirus*. HC‐Pro is capable of performing multiple important roles during viral infection, such as sequestering siRNAs and hijacking AGO1 to enhance the stability of the viral particle (Pollari et al., 2020; Valli et al., 2018).

While silencing suppressors have been well studied in plant viruses (Csorba et al., 2015; Lopez‐Gomollon & Baulcombe, 2022), they are only beginning to be identified and characterized in nonviral microbes such as plant‐pathogenic bacteria, fungi, and oomycetes (Qiao et al., 2013; Vetukuri et al., 2017).

Gene silencing and suppression of silencing involve trans‐kingdom movement of sRNAs (Kong et al., 2022). Trans‐kingdom movement of sRNAs is the transfer of sRNAs from one organism to another organism in a different evolutionary kingdom (Weiberg et al., 2013; Zhang et al., 2016). Once inside their target, these trans‐kingdom sRNAs hijack the host's silencing machinery to target key features of host defence. The role of plant trans‐kingdom sRNAs, particularly siRNAs, in moderating plant defence against viral and nonviral pathogens is beginning to be elucidated (Kong et al., 2022).

**FIGURE 2:**
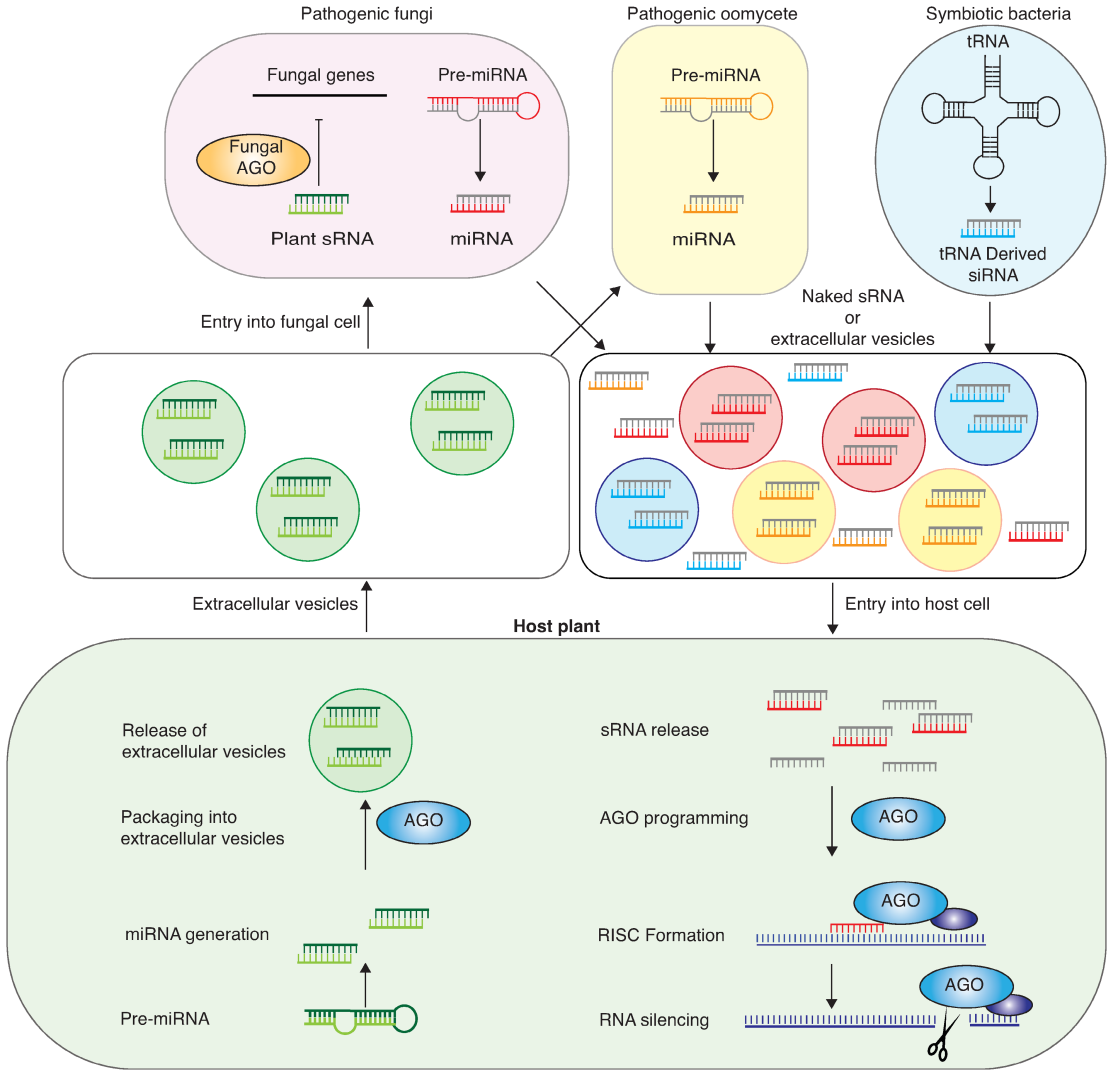
Trans‐kingdom movement of small RNAs from microbes to plants and from plants to microbes. Some plant small RNAs are packaged by AGO1 and other proteins into exosome‐like extracellular vesicles. These small RNAs are delivered into pathogen cells and silence pathogen genes likely by hijacking microbial AGO proteins. Several pathogens and symbionts generate small RNAs that move trans‐kingdom into plant cells by a mechanism that is not clear. However, it may involve exosome‐like extracellular vesicles or naked small RNAs. Microbe‐derived small RNAs use the plant cellular machinery to silence host genes.

Recent results show that pathogens send trans‐kingdom sRNAs to plants to disrupt, suppress, or modulate gene silencing in order to establish infection and enhance pathogenicity. Furthermore, plant symbionts such as rhizobia and mycorrhizal fungi also alter plant gene silencing pathways to establish symbiosis (Figure 2). Failure to interfere with host gene silencing results in reduced colonization by both pathogens and symbionts (Gui et al., 2022; Ren et al., 2019; Wong‐Bajracharya et al., 2022). In this review, we summarize the latest findings on how pathogenic and nonpathogenic microbes use trans‐kingdom sRNAs and silencing suppressors to induce and suppress gene silencing in plants.

## 2 TRANS‐KINGDOM sRNAs AND GENE SILENCING BY FUNGI AND OOMYCETES

The roles of exosome‐like extracellular vesicles in trans‐kingdom movement of sRNAs have been nicely summarized by several reviews (Borniego & Innes, 2023; Liu et al., 2022). Movement of sRNAs from pathogens to plants probably occurs through exosome‐like extracellular vesicles. Some plant sRNAs move into plant pathogens via exosome‐like extracellular vesicles (Cai et al., 2018). These vesicles are loaded with RNAs and RNA‐binding proteins, such as AGO1, and transported into pathogen cells (He et al., 2021). Extracellular vesicles are also involved in cross‐kingdom movement of sRNAs from parasitic nematodes into mammalian hosts (Buck et al., 2014; Duguet et al., 2020).

Virulent trans‐kingdom sRNAs were first described in the fungal pathogen *Botrytis cinerea*. sRNAs from *B. cinerea* are predicted to target up to 73 different genes in both *Arabidopsis thaliana* and *Solanum lycopersicum* (Weiberg et al., 2013). Notable target genes include *MPK1* and *MPK2*, which play a role in senescence and plant immunity (Zhang et al., 2020). One specific sRNA, *Bc*‐siR37, was shown to target multiple WRKY transcription factors in *Arabidopsis* (Wang et al., 2017). In transgenic *Arabidopsis* plants that ectopically express these sRNAs, resistance to infection by *B. cinerea* is greatly reduced, which demonstrates the role of trans‐kingdom sRNAs in suppressing host immunity during infection by *B. cinerea* (Wang et al., 2017). Interestingly, a functioning plant AGO1 is necessary for this silencing to occur (Wang et al., 2017). This shows not only that sRNAs are being sent trans‐kingdom to silence host genes, but also that endogenous plant AGO1 is hijacked to execute silencing.

Another pathogen shown to utilize trans‐kingdom sRNAs is the fungus *Rhizoctonia solani* (Meng et al., 2021). In maize, a total of 58 different genes were predicted to be targets of *R. solani*'s sRNAs. Some of these sRNAs were shown to target maize genes during the infection process to interfere with host immunity (Lee et al., 2003).

In *Fusarium graminearum*, the causal agent of Fusarium head blight, *Fg‐sRNA1*, an sRNA, was shown to suppress plant defence by targeting and silencing a gene encoding a chitin elicitor binding protein, CEBiP (Jian & Liang, 2019). CEBiPs are important components of resistance against fungal pathogens (Kaku et al., 2006; Tanaka et al., 2010). Silencing of *CEBiP* by Fg‐sRNA1 enhanced invasion by *F. graminearum* while also reducing plant resistance (Jian & Liang, 2019).

*Verticillium dahliae* also uses sRNA as effectors during its infection of *Arabidopsis* plants. These sRNAs hijack the plant's AGO1 and AGO2 proteins and use them to target and silence plant genes involved in resistance and successfully establish infection (Wang et al., 2016).

*Puccinia striiformis* f. sp. *tritici*, a fungal pathogen of wheat, has also been shown to generate sRNAs capable of silencing plant genes. The wheat pathogenesis‐related (*PR2*) gene *SM638* encodes a β‐1,3‐glucanase and is a target of the fungal sRNA Pst‐milR1. Deletion of the Pst‐milR1 precursor prevents infection of wheat plants by *P. striiformis*. Furthermore, *SM638* knockdown wheat mutants are susceptible to a *P. striiformis* isolate normally unable to infect the wheat cultivar. It was also shown that Pst‐milR1 does not target homologous *PR2* genes in wheat, highlighting the target specificity of certain trans‐kingdom sRNAs (Wang et al., 2017).

During infection of apple trees by the fungal pathogen *Valsa mali*, two genes encoding receptor‐like kinases, *MdRLKT1* and *MdRLKT2*, are targeted by the fungus' sRNA vm‐milR1 (Xu et al., 2022). Both genes are necessary for host resistance to *V. mali*. Vm‐milR1 deletion mutants are significantly less effective at infecting apple twigs than the wild‐type fungal strain, demonstrating the role of the sRNA in establishing successful infection (Xu et al., 2022).

sRNAs have also been described in oomycete pathogens (Table 1). Mechanistically, there is no characterized difference between sRNAs produced by fungi and those produced by oomycetes except for the sequences they target (Table 1). In *Hyaloperonospora arabidopsidis*, at least 34 sRNAs were predicted to target and silence *Arabidopsis* genes involved in host immunity. Just like fungal sRNAs, *H. arabidopsidis* sRNAs also use the host plant's AGO proteins to induce gene silencing in the host and enhance virulence (Dunker et al., 2020).

**TABLE 1:** Trans‐kingdom movement of small RNAs from pathogens and symbionts into plant cells.

Microbe	Host	siRNA length (nt)	Predicted host targets	Host argonaute effector	Reference
Pathogens					
*Botrytis cinerea*	*Arabidopsis thaliana*	20–35	152	AtAGO1	Weiberg et al. (2013)
	*Solanum lycopersicum*	20–35	163	Unknown	Weiberg et al. (2013)
*Rhizoctonia solani*	*Zea mays*	18–30	58	Unknown	Meng et al. (2021)
*Fusarium graminearum*	*Triticum aestivum*	18	264	Unknown	Jian and Liang (2019)
*Verticillium dahliae*	*A. thaliana*	20–35	378	AtAGO1, AtAGO2	Wang et al. (2016)
*Puccinia striiformis* f. sp. *tritici*	*T. aestivum*	19–30	4	Unknown	Wang et al. (2017)
*Fusarium oxysporum* f. sp. *lycopersici*	*S. lycopersicum*	23	1	SlyAGO4a	Ji et al. (2021)
*Valsa mali*	*Malus domestica*	18	184	Unknown	Xu et al. (2022)
*Hyaloperonospora arabidopsidis*	*A. thaliana*	19–30	34	AtAGO1	Dunker et al. (2020)
*Phytophthora infestans*	*Solanum tuberosum*	18–24	6,846	Unknown	Hu et al. (2022)
Symbionts					
*Bradyrhizobium japonicum*	*Glycine max*	18–24	52	GmAGO1b	Ren et al. (2019)
*Pisolithus microcarpus*	*Eucalyptus grandis*	20–24	19	Unknown	Wong‐Bajracharya et al. (2022)

*Phytophthora infestans*, the causal agent of potato late blight, is another oomycete capable of deploying pathogenic trans‐kingdom sRNA. Its sRNA miR8788 targets the gene *StABH1*, which encodes an α/β hydrolase localized to the plasma membrane that is essential for plant defence against *P. infestans* (Hu et al., 2022). miR8788 is the only microRNA that has been characterized so far in *P. infestans*. It has been found with an intact pre‐miR8788 sequence in European plant samples collected in 1846 and 1877. As plant pathogens commonly adapt new virulence mechanisms and replace old ones, it is fascinating to see that this microRNA has remained highly conserved in *P. infestans* for so long, which points to its critical role in virulence of *P. infestans* (Hu et al., 2022).

## 3 TRANS‐KINGDOM sRNAs AND GENE SILENCING BY PLANT SYMBIONTS

Plant symbionts need to slip through host defences to properly establish symbiosis. This is primarily achieved by the use of lipochito‐oligosaccharides (LCOs) produced by the microbe (Limpens et al., 2015). Rhizobial LCOs, known as nodulation (Nod) factors, reduce innate plant immunity by decreasing the concentration of MAMP receptors (Liang et al., 2013). Even plants not able to form symbiosis with *Rhizobium*, such as *Arabidopsis*, are susceptible to these Nod factors. Because trans‐kingdom sRNAs weaken plant immunity, they are another tool that symbionts use to subvert plant defences and establish symbiosis.

As prokaryotic organisms, bacteria do not possess the sRNA silencing machinery that is conserved in eukaryotes. Thus, no pathogenic bacterium has been shown to utilize trans‐kingdom sRNAs when infecting plants. However, rhizobia have been found to interact with *Glycine max* through trans‐kingdom tRNA‐derived fragments (tRFs) (Ren et al., 2019). These tRFs are generated from tRNA and are capable of associating with AGO proteins (Kumar et al., 2014). In rhizobia–legume symbiosis, this unique class of sRNAs associates with and hijacks *GmAGO1* to induce silencing of antinodulation genes. This results in a significant increase in both the number and size of root nodules. Rhizobia also utilize the type III effector Bel2‐5 to forcibly promote nodulation in a similar manner to these trans‐kingdom sRNAs (Ratu et al., 2021), further demonstrating the functional overlap between trans‐kingdom sRNAs and traditional protein effectors.

Fungi also form important symbiotic relationships with plants. Perhaps the most well‐known forms of plant–fungus symbioses are mycorrhizae. The ectomycorrhizal fungus *Pisolithus microcarpus* has been found to send at least 11 microRNAs into the host tree, *Eucalyptus grandis* (Wong‐Bajracharya et al., 2022). The *P. microcarpus* miRNA *Pmic_miR‐8* was shown to be transported to the root system of *E. grandis*. Once inside root cells, it promotes establishment of ectomycorrhizal symbiosis and increases the development of the Hartig net. This increase in fungal colonization appears to be a result of *Pmic_miR‐8* targeting multiple NLR transcripts encoded by the host (Wong‐Bajracharya et al., 2022).

While trans‐kingdom sRNAs have not been directly confirmed in arbuscular mycorrhizae, there is evidence that they might play a critical role in this symbiosis as well. Analysis of sRNA reads from the arbuscular mycorrhizal fungus *Gigaspora margarita* shows that it generates sRNAs that target genes in *Medicago truncatula*. A total of 297 different *M. truncatula* mRNAs were shown to be potential targets of *G. margarita* sRNAs (Silvestri et al., 2020). Further research is needed to confirm if these sRNAs actually transfer over and play a role in symbiosis.

## 4 SILENCING SUPPRESSION BY PLANT‐PATHOGENIC MICROBES

Pathogens have evolved a diverse suite of effector molecules to overcome plant defence and get nutrients and/or resources from their host plants. Plant viruses encode a critical group of effectors that suppress gene silencing. Virus‐encoded silencing suppressors disrupt both antiviral and endogenous gene RNA silencing, thus interfering with normal plant growth and antiviral immunity. Silencing suppressors are capable of targeting the gene silencing at each step of the pathway, including preventing sRNA biogenesis (Landeo‐Ríos et al., 2016), sequestration of sRNAs to prevent their association with AGO proteins (Hamera et al., 2012; Kontra et al., 2016; Pérez‐Cañamás & Hernández, 2015), degradation of AGO proteins (Csorba et al., 2010; Karran & Sanfaçon, 2014), and inhibition of silencing amplification (Guo et al., 2013; Li et al., 2014).

While traditionally associated with plant viruses, silencing suppressors have now been identified in nonviral pathogens (Table 2). So far, these nonviral silencing suppressors have been found in pathogenic fungi, bacteria, and oomycetes. Some evidence also suggests that plant‐pathogenic nematodes are capable of suppressing gene silencing. However, no specific effector proteins capable of suppression have yet been identified (Walsh et al., 2017). Nonviral silencing suppressors, like their viral counterparts, function by disrupting one or more components of the host plant's gene silencing pathway (Figure 1).

**TABLE 2:** Silencing suppressors of nonviral plant pathogens.

Pathogen	Host	Suppressor	Suppression mechanism	Deletion mutant	Reference
Fungi					
*Puccinia graminis*	*Nicotiana benthamiana*	PgtSR1	Prevents R gene‐triggered hypersensitive response	Unknown	Yin et al. (2019)
*Verticillium dahliae*	*Arabidopsis thaliana*, *N. benthamiana*	VdSSR1	Interferes with the nuclear export of AGO1–miRNA complexes reducing plant‐to‐fungi trafficking of trans‐kingdom siRNA	Reduced virulence	Zhu et al. (2022)
Oomycetes					
*Phytophthora sojae*	*N. benthamiana*	PSR1	Impairs assembly of microRNA‐processing complexes and causes reduced abundance of siRNAs	Unknown	Qiao et al. (2013, 2015)
*P. sojae*	*N. benthamiana*	PSR2	Interferes with biogenesis of secondary siRNAs (tasiRNAs). May inhibit salicylic acid defence pathway	Reduced virulence	Qiao et al. (2013); Xiong et al. (2014); Hou et al. (2019); Gui et al. (2022)
*Phytophthora infestans*	*N. benthamiana*	Pi14054	Unknown	Unknown	Vetukuri et al. (2017)
Bacteria					
“*Candidatus* Phytoplasma tritici”	*N. benthamiana*	SWP16	Inhibits biogenesis of some miRNAs	Unknown	Wang et al. (2021)
*Pseudomonas syringae* pv. *tomato*	*A. thaliana*	AvrPto	Unknown	Unknown	Navarro et al. (2008)
		hopT1	Unknown	Unknown	Navarro et al. (2008)
		hopN1	Unknown	Unknown	Navarro et al. (2008)

The first nonviral silencing suppressors were identified in the bacterial pathogen *Pseudomonas syringae* pv. *tomato* and were shown to disrupt normal accumulation of certain specific microRNAs in *Arabidopsis* (Navarro et al., 2008). One of these identified microRNAs, miR393, is relevant in pattern‐triggered immunity (PTI). Another bacterial silencing suppressor, SWP16, has been identified in the wheat blue dwarf phytoplasma and shown to reduce miRNA accumulation in *Arabidopsis* (Wang et al., 2021). When inserted into the potato virus X genome, SWP16 improved virulence and enhanced disease symptoms. Of the currently identified bacterial silencing suppressors, all seem to reduce accumulation of sRNAs by some mechanism (Table 2). Preventing accumulation and synthesis of sRNAs is also a common strategy utilized by viral silencing suppressors, such as P1b of cucumber vein yellowing virus (Valli et al., 2011).

The silencing suppressors of oomycetes are the best‐studied suppressors of all the nonviral pathogens. Similar to bacterial suppressors, oomycete suppressors appear to target the synthesis and accumulation of sRNAs (Table 2). The first oomycete silencing suppressors to be characterized are suppressors of RNA silencing 1 and 2, PSR1 and PSR2, from *Phytophthora sojae* (Qiao et al., 2013). PSR1 binds to PSR1‐Interacting Protein 1 (PINP1), a pre‐mRNA splicing factor, preventing it from binding to pre‐mRNA and causing alternative splicing of the mRNA. This blocks the production of proteins and sRNAs that are critical for plant immunity and promotes infection by *P. sojae* (Gui et al., 2022). PSR2, on the other hand, interacts with dsRNA‐binding protein 4 (DRB4) and interferes with its role in the biogenesis of sRNAs that are necessary for the plant to protect itself against *P. sojae* (Hou et al., 2019). Knocking out *PSR2* in *P. sojae* greatly reduces the pathogen's virulence. When transgenically expressed in *Arabidopsis*, both PSR1 and PSR2 were shown to reduce sRNA biogenesis (Qiao et al., 2013). Homologues of PSR2 have been found in at least eight other *Phytophthora* species, including *P. infestans* (de Vries et al., 2017; Qiao et al., 2013; Xiong et al., 2014). Another novel silencing suppressor, Pi14054, was also identified in *P. infestans*, but little is known about its mechanism of suppression (Vetukuri et al., 2017). The identification of multiple silencing suppressors among *Phytophthora* species implies that RNA silencing is critical for plant defence against *Phytophthora* and that it must be overcome by the pathogen.

Some fungal pathogens also possess effectors that function as silencing suppressors necessary for successful infection of host plants (Table 2). The first identified fungal silencing suppressor is PgtSR1 found in *Puccinia graminis* (Yin et al., 2019). PgtSR1 alters the abundance of sRNAs involved in plant defence and blocks the hypersensitive response that is normally triggered by R proteins upon detection of a pathogen, thus impeding both basal defences and effector‐triggered immunity (Yin et al., 2019). How PgtSR1 exactly interferes with sRNAs and the hypersensitive response remains unknown. It is worth noting that several viral silencing suppressors trigger the host's hypersensitive response. These suppressors include P0 of turnip yellows virus (Wang et al., 2015), P19 of tomato bushy stunt virus (Angel & Schoelz, 2013), and nonstructural protein S (NSs) of tomato spotted wilt virus (De Ronde et al., 2013). It is not clear if any nonviral silencing suppressor can elicit the hypersensitive response. Homologues of PgtSR1 have also been identified in other fungi in silico but have not yet been characterized (Nandety et al., 2022).

In *V. dahliae*, the silencing suppressor VdSSR1 works by sequestering off ALY proteins that are required for the nuclear export of AGO1 proteins (Zhu et al., 2022). Without AGO1, cytoplasmic sRNA levels are greatly reduced in the plant cell, including those of miR159 and miR166, which were shown to move trans‐kingdom from cotton and *Arabidopsis* plants to *V. dahliae* cells to silence virulence genes. Knocking out *VdSSR1* results in a significant reduction of virulence in *Arabidopsis* and cotton plants. This effector, perhaps more than any other silencing suppressor, emphasizes the critical role of sRNA cross‐talk between microbes and plants. It is possible that *V. dahliae* has evolved a silencing suppressor that acts to pre‐emptively stop trans‐kingdom miRNAs that would target its own virulence genes. Interacting with AGO proteins, especially AGO1, is another common mechanism of several viral silencing suppressors such as P0 of pea enation mosaic virus‐1 (Fusaro et al., 2012), P25 of potato virus X (Chiu et al., 2010), and CP from tomato ringspot virus (Karran & Sanfaçon, 2014). As a core component of RISC, AGO proteins are a logical target for disruption by silencing suppressors. So far, VdSSR1 is the only nonviral silencing suppressor that has been shown to directly interfere with an AGO protein (Table 2).

## 5 CONCLUSIONS

Gene silencing has been well characterized as a core component of plant antiviral immunity.

Virus‐derived sRNAs are critical determinants of antiviral immunity mediated by gene silencing and virus‐encoded silencing suppressors are critical determinants of virus pathogenicity. The discovery of trans‐kingdom movement of sRNAs into plants from pathogens and symbionts and vice versa puts the role of gene silencing in a new light. Recent research has shown that gene silencing also plays a crucial role in mediating defence responses against nonviral microbes. Some fungal and oomycete pathogens send sRNAs capable of hijacking the plant gene silencing machinery to disrupt immunity. Symbiotic partners of plants, such as mycorrhizal fungi and rhizobia, also send out trans‐kingdom sRNAs to disarm their host's innate immune system and establish symbiosis. The mechanisms of trafficking sRNAs from microbes to host plants remain poorly understood. It is likely that some form of extracellular vesicles are employed in the process (Figure 2). Plant‐pathogenic microbes also produce gene silencing suppressors that disrupt sRNA biogenesis and/or activity in the host and impede its ability to establish a defence response. It is also possible that these silencing suppressors interfere with sRNAs that move trans‐kingdom into their cells from the host plant.

## 6 FUTURE DIRECTIONS

Trans‐kingdom sRNAs are common among plant‐associated microbes, pathogenic or symbiotic. Their function is to weaken the plant's immunity to enhance pathogenicity or establish symbiosis. Identifying new trans‐kingdom sRNAs is an important step in understanding how these microbes circumvent plant immunity. Because trans‐kingdom sRNAs naturally target genes with important resistance functions, they can potentially be used to identify core components of plant immunity.

The role of silencing suppressors in the interaction between plants and their associated microbes is still not fully understood. While they appear to act primarily as effector proteins that disrupt immunity, they may very well play a more generic role in regulating the microbes' own silencing pathways. Alternatively, or in addition, they may remain inside the microbe and work to deactivate trans‐kingdom sRNAs being sent over by plants. In this case, they would act as a shield against harmful trans‐kingdom sRNAs. Some pathogens, such as *P. infestans*, use both silencing suppressors and trans‐kingdom pathogenic sRNAs. At first glance, these two systems would seem at odds with each other, where silencing suppressors disable the machinery that is necessary for the pathogenic siRNA to silence host genes. One possible explanation for this may be that these different mechanisms of virulence occur at different stages of infection or act in different physical locations. Potentially, trans‐kingdom sRNAs down‐regulate core developmental or immunity genes in host plants, while silencing suppressors interfere with sRNAs getting into the pathogen from the plant. It would be informative to determine if silencing suppressors from nonviral pathogens bind siRNAs that are of plant origin. This would further clarify the importance of plant trans‐kingdom RNA as a potent method of counterattack and demonstrate a defensive role that pathogens may be using against plant siRNA.

## CONFLICT OF INTEREST STATEMENT

The authors declare no conflict of interest. The founding sponsors had no role in the design of the study, in the collection, analyses, or interpretation of data, in the writing of the manuscript, or in the decision to publish the results.

## Data Availability

Data sharing is not applicable to this article as no new data were created or analysed.
